# Effects of host plant on life-history traits in the polyphagous spider mite *Tetranychus urticae*

**DOI:** 10.1002/ece3.1554

**Published:** 2015-07-14

**Authors:** Cassandra Marinosci, Sara Magalhães, Emilie Macke, Maria Navajas, David Carbonell, Céline Devaux, Isabelle Olivieri

**Affiliations:** 1 ISEM, Institut des Sciences de l’Evolution Montpellier, UMR 5554 (Université de Montpellier/CNRS/IRD)Place Eugène Bataillon, 34095, Montpellier Cedex 05, France; 2 CE3C, Centre for Ecology, Evolution and Environmental Sciences, Faculdade de Ciências da Universidade de LisboaEdificio C2, 3° Piso, Campo Grande, P-1749016, Lisbon, Portugal; 3 Laboratory Aquatic Biology, KU Leuven KulakE. Sabbelaan 53, 8500, Kortrijk, Belgium; 4 INRA UMR CBGP (INRA/IRD/Cirad/Montpellier SupAgro)Campus International de Baillarguet, CS 30016, F-34988, Montferrier-sur-Lez Cedex, France; 5 CNRS, Institut des Sciences de l’Evolution Montpellier, UMR 5554 (Université de Montpellier/CNRS/IRD)Bât. 22, Place Eugène Bataillon, 34095, Montpellier Cedex 05, France

**Keywords:** Adaptation, host shift, maternal effects, plant acceptance, plant–herbivore interactions

## Abstract

Studying antagonistic coevolution between host plants and herbivores is particularly relevant for polyphagous species that can experience a great diversity of host plants with a large range of defenses. Here, we performed experimental evolution with the polyphagous spider mite *Tetranychus urticae* to detect how mites can exploit host plants. We thus compared on a same host the performance of replicated populations from an ancestral one reared for hundreds of generations on cucumber plants that were shifted to either tomato or cucumber plants. We controlled for maternal effects by rearing females from all replicated populations on either tomato or cucumber leaves, crossing this factor with the host plant in a factorial design. About 24 generations after the host shift and for all individual mites, we measured the following fitness components on tomato leaf fragments: survival at all stages, acceptance of the host plant by juvenile and adult mites, longevity, and female fecundity. The host plant on which mite populations had evolved did not affect the performance of the mites, but only affected their sex ratio. Females that lived on tomato plants for circa 24 generations produced a higher proportion of daughters than did females that lived on cucumber plants. In contrast, maternal effects influenced juvenile survival, acceptance of the host plant by adult mites and female fecundity. Independently of the host plant species on which their population had evolved, females reared on the tomato maternal environment produced offspring that survived better on tomato as juveniles, but accepted less this host plant as adults and had a lower fecundity than did females reared on the cucumber maternal environment. We also found that temporal blocks affected mite dispersal and both female longevity and fecundity. Taken together, our results show that the host plant species can affect critical parameters of population dynamics, and most importantly that maternal and environmental conditions can facilitate colonization and exploitation of a novel host in the polyphagous *T. urticae*, by affecting dispersal behavior (host acceptance) and female fecundity.

## Introduction

Plants and herbivores exert strong selective pressures upon each other (Schoonhoven et al. 2005), resulting in a coevolutionary arms race (Ehrlich and Raven 1964; Kant et al. 2008). To cope with attacks by herbivores, plants have evolved physical defenses, such as glandular trichomes, and a large diversity of chemical defenses (Fritz and Simms 1992; Schoonhoven et al. 2005; Wise and Rausher 2013). Conversely, herbivores have evolved mechanisms to cope with these defenses, by escaping detection by host plants or suppressing plant defenses (Schoonhoven et al. 2005; Kant et al. 2008; Sarmento et al. 2011). The herbivore ability to get around host plant defenses is particularly important for polyphagous species, which can feed and reproduce on several plant families (Schoonhoven et al. 2005), relative to monophagous species, which are specialized on a few closely related plant species expected to carry similar defenses. For polyphagous species that can frequently be exposed to new hosts, colonizing a new host plant species often involves tolerating or resisting defenses specific to the new host plant, and thus quickly responding to diverse and possibly strong selection. Responses of herbivore populations to environmental changes, such as a host plant shift, can range from local extinction to adaptation. A related question is how and by which mechanisms can populations of polyphagous herbivores adapt and exploit many host plant species. Addressing this question is not only exciting from an evolutionary perspective, for example, to understand local adaptation processes, but can also provide crucial information for the management of crop pests.

To test for local adaptation of herbivore populations to a given host plant species, a common practice is to compare their fitness components (e.g., life-history traits) on this plant species with those displayed by other herbivore populations that have evolved on a different plant species (Kawecki and Ebert 2004). It is expected that local genotypes have a higher fitness on their (local) host plant species than do genotypes from a different host plant species. As emphasized by Kawecki and Ebert (2004), experimental evolution is a valuable way to complement research on natural populations using replicated populations in controlled environments and by allowing direct tests for adaptation. This approach allows researchers to establish causality of evolutionary processes and adaptation patterns (Kawecki et al. 2012a,b*;* Magalhães and Matos 2012), which is impossible with natural populations. Yet, experimental evolution has barely been used to study the adaptation of herbivores to new host plant species (but see Gould 1979; Fry 1990; Bolter and Jongsma 1995; Agrawal 2000; Fricke and Arnqvist 2007; Magalhães et al. 2007; Kojima et al. 2010; Fox et al. 2011; Fellous et al. 2014). Moreover, the few studies that used this approach were limited in the number of traits measured and by the fact that plasticity and dispersal processes were usually not simultaneously analyzed.

*Tetranychus urticae* Koch (Acari: Tetranychidae) is a highly polyphagous spider mite that feeds on more than 1100 plant species, of which over 100 are important agricultural crops (http://www1.montpellier.inra.fr/CBGP/spmweb/). Recent studies revealed that adaptation of this herbivore to one host plant could facilitate exploitation of other host plant species and therefore broaden its host range (Magalhães et al. 2009; Fellous et al. 2014). Further, adaptation to a new host plant for this species did not always result in its decreased performance on the original host (Gould 1979; Fry 1990; Agrawal 2000; Magalhães et al. 2009). After 15 generations, mite populations evolving and tested on detached leaves of a novel host species (tomato or pepper) performed better on their host than did populations evolving on leaves of another host (cucumber), indicating the occurrence of local adaptation (Magalhães et al. 2007), and adaptation to a specific host plant (here the tomato leaves). In this study, we investigated the responses of *T. urticae* to a host shift, using mite populations maintained on entire plants and tested on detached leaves. As in Magalhães et al. (2007), we used populations maintained on cucumber plants in our laboratory. We measured survival, fecundity, changes in the sex ratio (proportion of males), and the host plant acceptance (Via 1990), which can inform on the dispersal behavior of *T. urticae*. Tests for adaptation were performed on detached leaves to measure life-history traits and to monitor the life cycle of single individuals.

## Material and Methods

### The experimental populations

In May 1994, *T. urticae* mites were collected from a population maintained on cucumber plants (*Cucumis sativus*, variety: Ventura) in a greenhouse in Pijnacker, the Netherlands, then kept on the same variety in a climate chamber at the University of Amsterdam. In September 2007, after they had evolved for circa 480 generations, approximately 5000 mites were sampled from the Amsterdam population to create two populations on cucumber plants at the University of Montpellier, hereafter called “ancestral populations”.

In February 2011, eight independent populations were created from a mixture of the two ancestral populations: four populations each containing 400 females were transferred on the same variety of cucumber plants (hereafter called C*i*, for population *i*) and the other four populations containing, respectively, 400, 400, 260, and 220 females were transferred to tomato plants (*Solanum lycopersicum* species, variety Olympe HF1; hereafter called T*i*). One month later (circa 2 generations), because T1 and T2 experienced a demographic bottleneck, 400 mite females (a mixture of one hundred mites of each C*i* population) were added to each of these populations. All populations were maintained under the same conditions in a single climate chamber at 25 ± 1°C with a photoperiod of 14L: 10D and were in different boxes isolated from one another by water (to prevent mites from dispersing). Two to four new plants, depending on the number of old plants, were added every week to each population while old plants were removed. Plants used to maintain mite populations and test for adaptation and maternal effects (see below) were grown from seeds placed during one and a half months in an isolated and herbivore-free room controlled for temperature (23 ± 1°C) and photoperiod (12L:12D).

### Tests for adaptation and maternal effects

When life-history traits were measured, the C populations had evolved on cucumber plants (in different countries/conditions) for circa 594 generations (17 years and 9 months), and the T populations had evolved for circa 570 generations (16 years and 9 months) on cucumber and 24 generations (1 year) on tomato plants. Two temporal blocks of the experiment presented below were performed in January and February 2012; hence, populations had evolved two more generations in the second block compared to the first block. Hereafter the selection regime (SRT for the Selection Regime Tomato, and SRC for the Selection Regime Cucumber) refers to the host plant species (T and C, respectively) on which the populations had evolved the year previous to the experiment.

To study adaptation and maternal effects, mites from each population spent one generation in one of two distinct maternal environments, consisting of either detached cucumber leaf fragments or detached tomato leaves (hereafter the term “leaf” is used to refer to both maternal environments). Thirty adult females were haphazardly sampled from each of the C and T populations and put on either tomato or cucumber leaves of similar sizes (circa 16 cm²) and then put in plastic boxes covered with water-saturated cotton to both limit mite dispersal and ensure humidity and thus persistence of leaves. Twenty adult females produced by this first generation were then haphazardly sampled, during 1 day only, from each population in each maternal environment, and put on fresh leaves of the same species as the one experienced by their mothers. Note that because of high mortality in the second block, only thirteen and fifteen adult females for the C2 and C3 populations, respectively, were sampled from the tomato maternal environment. We let females oviposit in groups for 1 day, and twenty-four of the resulting eggs were haphazardly sampled from each population and each maternal environment. Eggs were individually put on a tomato leaf fragment of circa 0.8 cm², hereafter called the testing environment, to measure life-history traits and other fitness components. As monitoring was performed every 2 days and eggs were laid less than 24 h before being isolated, the precision of measures for each trait is less than 2 days. Again, leaf fragments were placed on water-saturated cotton in several plastic boxes. Fresh leaf fragments were provided to individual mites every 4 days. Twenty-four boxes, each containing 16 individuals, were then haphazardly put in a climatic room at 25 ± 1°C with a photoperiod of 14L:10D.

We recorded the status of mites (presence or absence, and alive or dead) on the testing environment every 2 days. Once mites had reached adulthood, we could identify their sex and count the number of eggs produced by females. All emerged females remained virgin during the experiment. Of the 768 individuals monitored in the two blocks, we measured life-history traits for 712 individuals (Table1); fifty-six individuals were removed from the analyses because they were accidentally lost. This monitoring provided for each block the individual following measures: (1) host acceptance at both juvenile and adult stages (i.e., to stay in or leave the testing environment), (2) egg mortality, (3) juvenile mortality, (4) developmental time, that is, the time to reach maturity, (5) sex, (6) time spent as an adult in the testing environment, (7) dispersal time of dispersing juveniles and adults, that is, the time at which they left the testing environment, and (8) fecundity of females, that is, the total number of eggs produced by each female (Table1).

**Table 1:** Sample sizes at generation 24 for each life-history trait according to the selection regime (SRC and SRT for cucumber and tomato plants, respectively), the maternal environment (MEC and MET for cucumber and tomato leaves, respectively) and the block (B1 and B2)

	SRC				SRT				Total
	MEC		MET		MEC		MET		
	B1	B2	B1	B2	B1	B2	B1	B2	
Egg mortality	83	91	85	93	92	91	87	90	712
Juvenile acceptance	75	86	79	93	87	85	83	88	676
Juvenile mortality	70	83	75	86	86	83	79	83	645
Developmental time, Sex ratio and Adult acceptance	56	42	60	64	67	50	68	70	477
Time spent as an adult in the testing environment	56	42	57	64	66	50	66	70	471
Fecundity	44	34	47	44	56	40	62	60	387

All analyses were carried out using the R software (R Core Team, 2013). Analyses of life-history traits were performed by building general or generalized linear mixed models including as fixed effects the selection regime (SR, with two levels), the maternal environment (ME, with two levels), the block (B, with two levels), and the interaction between the selection regime and the maternal environment. The block was included as a fixed rather than a random effect because it strongly affected late mite stages. Replicated populations were included as random factors nested within the selection regime. Fixed and random factors were tested by likelihood ratio tests. For all response variables, the best models included a single estimate of the interpopulation variance independently of the selection regime (all *P* values >0.18). When analyzing developmental time, adult acceptance and time spent as adult in the testing environment, the factor sex (with two levels) was included in the models. For the analysis of the time spent as an adult in the testing environment and fecundity, the factor adult acceptance (with two levels) was included in the models. We considered binomial distributions for the numbers of dead eggs, dead juveniles, dispersing juveniles, dispersing adults, and males, and thus applied generalized linear mixed models when analyzing variation in these response variables. The time spent as as an adult in the testing environment, the log-transformed developmental time and the square-rooted fecundity were assumed to follow normal distributions, and we thus applied linear mixed models for the analysis of these response variables. All models were validated by checking their error structure.

## Results

### Selection regime

**Figure 1:**
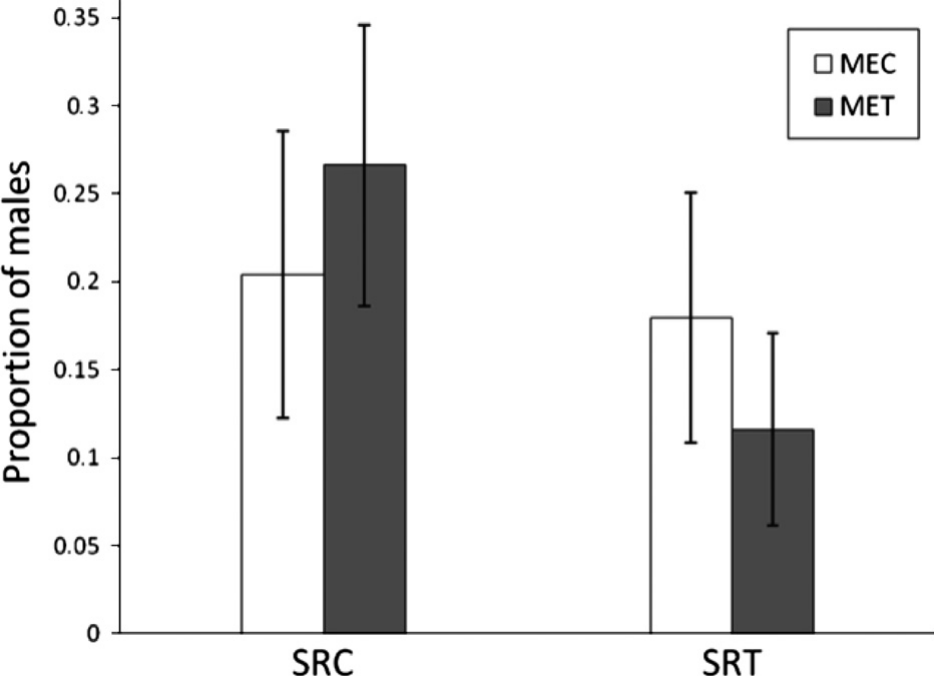
Sex ratio (proportion of males) of individuals that reached maturity as a function of the selection regime (SRC and SRT for cucumber and tomato, respectively) and the maternal environment (MEC and MET for cucumber and tomato, respectively).

After circa 24 generations of evolution, only the sex ratio of the populations differed between the selection regimes induced by the host plants: females from the populations maintained on tomato plants produced a lower proportion of sons (mean ± SE: 0.15 ± 0.03) than did females (0.24 ± 0.08) from the populations on cucumber plants, independently of the environment on which they laid eggs (Table2, Fig.1). Overall, we found no effect of the selection regime or its interaction with the maternal environment on any of the juvenile or adult traits (Tables2 and 3).

**Table 2:** Effects of the experimental design on sex ratio, egg mortality, juvenile acceptance, and juvenile mortality

Effects	Sex ratio		Egg mortality		Juvenile acceptance		Juvenile mortality	
	χ^2^	*P*-value	χ^2^	*P*-value	χ^2^	*P*-value	χ^2^	*P*-value
SR	**4.46**	**0.03**	0.21	0.65	1.82	0.18	1.77	0.18
ME	0.03	0.87	**4.38**	**0.04**	2.50	0.11	**18.51**	**<0.0001**
SR x ME	3.23	0.08	0.17	0.68	1.16	0.28	0.78	0.38
B	1.54	0.21	3.36	0.07	0.06	0.80	**15.94**	**<0.0001**

SR, selection regime; ME, maternal environment; B, block. χ^2^ and *P*-value are in bold when the *P*-value <0.05.

**Table 3:** Effects of the experimental design on adult acceptance, developmental time, time spent as an adult in the testing environment, and fecundity

Effects	Adult acceptance		Developmental time		Time spent as an adult in the testing environment		Fecundity	
	χ^2^	*P*-value	χ^2^	*P*-value	χ^2^	*P*-value	χ^2^	*P*-value
SR	1.08	0.30	0.68	0.41	0.42	0.51	0.03	0.87
ME	**8.19**	**0.004**	0.50	0.48	1.62	0.20	**8.93**	**0.003**
SR × ME	0.02	0.88	0.26	0.61	0.12	0.72	0.14	0.71
B	0.44	0.51	1.40	0.24	**30.59**	**<0.0001**	**73.5**	**<0.0001**
Sex	0.01	0.91	1.46	0.23	**6.21**	**0.01**	–	–
Adult acceptance status	–	–	–	–	**20.56**	**<0.0001**	–	–
Female acceptance status	–	–	–	–	–	–	**9.16**	**0.003**

SR, selection regime; ME, maternal environment; B, block. The ‘–’ indicates that the variable was not tested. χ^2^ and *P*-value are in bold when the *P*-value <0.05.

### Maternal environment

**Figure 2:**
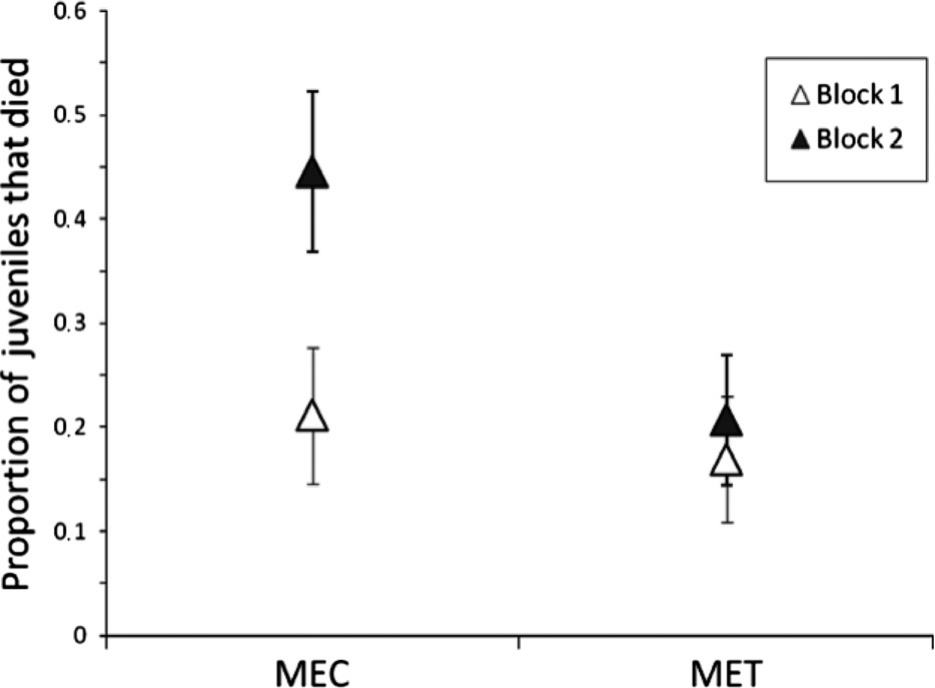
Proportion of dead juveniles in the testing environment as a function of the maternal environment (MEC and MET for cucumber and tomato, respectively) and the block (with white for B1 and black for B2); vertical lines correspond to four standard errors.

**Figure 3:**
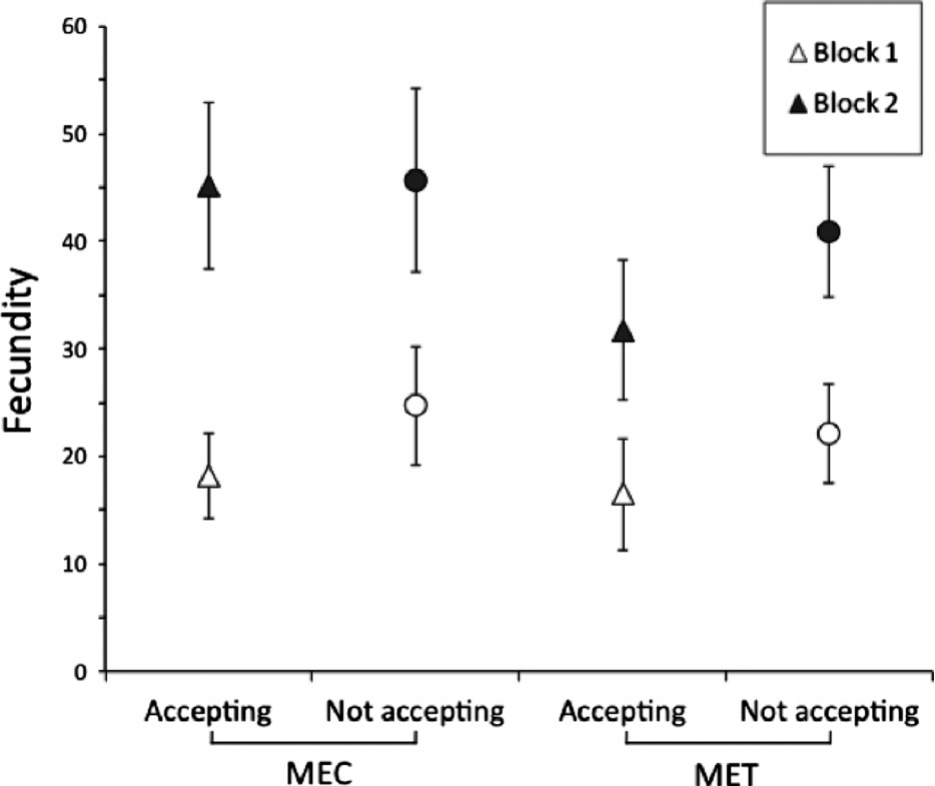
Fecundity of females in the testing environment as a function of the maternal environment (MEC and MET for cucumber and tomato, respectively), the female acceptance status (with circles for not accepting females and triangles for accepting females), and the block (with white for B1 and black for B2). Vertical lines correspond to four standard errors.

The maternal environment influenced mortality at both the egg and juvenile stages: offspring of females from the cucumber maternal environment had a higher mortality (0.07 ± 0.01 and 0.33 ± 0.03 for egg mortality and juvenile mortality, respectively) than offspring of females from the tomato maternal environment (0.03 ± 0.01 and 0.09 ± 0.02 for egg mortality and juvenile mortality, respectively). Note also that juvenile mortality was higher in the second block (0.33 ± 0.03) than it was in the first block (0.19 ± 0.02; Table2, Fig.2). As adults, individuals (males or females) accepted less the testing environment (acceptance: 0.43 ± 0.03) and produced (as females) fewer offspring (fecundity: 28.08 ± 1.54) if their mothers were reared on the tomato environment than if their mothers were reared on the cucumber environment (0.56 ± 0.03 and 31.34 ± 1.80 for adult acceptance and fecundity, respectively; Tables2 and 3, Fig.3). The maternal environment therefore acted in opposite directions on juvenile and adult fitness traits.

### Status of individuals

**Figure 4:**
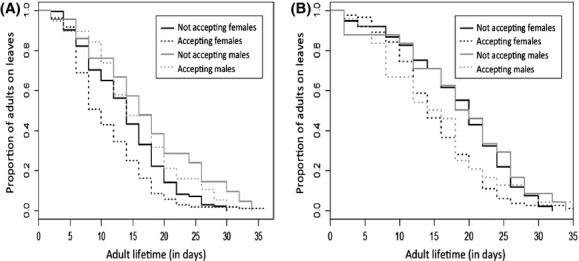
Proportion of live adults in the testing environment through days for blocks B1 (A) and B2 (B). The black lines hold for females, the gray lines for males, the solid lines for adults that accepted the testing environment, and the dotted lines for adults that did not.

The adult acceptance status*,* that is, the choice to stay in or disperse from the testing environment, the sex of the individuals and the block were relevant to describe the time spent as an adult on the testing environment. Independently of the selection regime and the maternal environment, male adults spent more time (16.77 days ± 0.92 days) in the testing environment than did females (14.56 ± 0.37; Table3, Fig.4) and also lived longer than did females (result not shown). Opposite patterns were observed for adult mites on the testing environment, depending on the blocks: adults dispersed at a younger age for the first block (age in days: 14.46 ± 0.65) than they did for the second block (18.57 ± 0.73), but they died older in the second block (age at death in days: 15.26 ± 0.70) than they did in the first block (11.75 ± 0.59; Table3, Fig.4).

Female fecundity was influenced by their acceptance status and by the block. Females that dispersed from the testing environment produced more eggs (32.72 ± 1.67) than did females that accepted this environment (26.27 ± 1.62). The fecundity of females was also twice higher in the second block (40.53 ± 1.80) than in the first block (20.20 ± 1.21), probably because females lived longer in the second block than they did in the first one (Table3, Figs.3 and 4).

## Discussion

In this study, we investigated the evolution of demographic and behavioral traits in response to a host plant shift in populations of a polyphagous mite. The fitness components of these populations were measured while controlling for maternal effects. We did not find signals of adaptation of the populations to the novel host, but detected strong maternal effects and a difference in sex ratio between host plants on which the populations had evolved.

### Observed adaptive changes after the host shift

Our experiment reveals that 1 year (circa 24 generations) of evolution of *T. urticae* on tomato plants did not affect their juvenile or adult traits when measured on tomato leaves. As *T. urticae* is an extreme generalist, populations may have been already adapted to both tomato and cucumber plants. However, Magalhães et al. (2007), who used the same ancestral population of *T. urticae* and also controlled for maternal effects*,* detected adaptation after 15 generations for mite populations that had evolved and were tested on tomato leaves. These contrasting results may stem from the material used to maintain the populations. Kavousi et al. (2009) detected different responses in demographic traits in *T. urticae* when measured on leaf disc versus whole leaves. Adaptation to plants versus detached leaves may thus imply different levels of stress and environmental variation require different adaptive processes. Although detached leaves are able to produce chemical defenses (Dicke and Dijkman 1992), much of the plant response to herbivores is systemic and thus absent in detached leaves. To measure accurately adaptation to novel host plants, populations should be maintained and tested on entire plants, and maternal effects should be controlled for.

The present study also shows that the host plant on which the population has evolved influences the sex ratio, measured as the proportion of sons, with females maintained on tomato plants producing a lower proportion of sons than females maintained on cucumber plants. The same effect was observed when sex ratio was measured directly in evolving populations, maintained on either cucumber or tomato plants (unpublished data). The low proportion of males in the populations is thus consistent with the observed and lower density of individuals on tomato plants compared to cucumber plants. The low density of mites on tomato plants can result from several non-exclusive mechanisms: the lack of adaptation to the novel host plant, the low resources provided to mites by tomato plants (as suggested by our personal observations on female fecundity and survival), and the increased dispersal of adult individuals. For *T. urticae,* females have been shown to be the dominant dispersing sex (Kennedy and Smitley 1985; Bitume et al. 2011); we, however, did not detect differences between male and female dispersal behavior as they left the testing environment equally (circa about 50% of adult mites). The low proportion of males in some populations is also not consistent with the idea that tomato plants may provide fewer resources to females compared to cucumber plants. In *T. urticae* indeed, males result from smaller eggs than do females (Macke et al. 2011), and may thus be less costly to produce in a poor environment.

### Maternal effects increase offspring fitness on the novel host plant

Independently of the host plant species on which the populations were reared, we found that the survival of juveniles from mothers that lived on tomato leaves was near twice higher than was the survival of offspring from mothers that lived on cucumber leaves, suggesting that the tomato was a better previous environment than the cucumber was to then exploit tomato host plant. It has been indeed found that insect individuals produced by mothers experiencing a low-quality environment have a lower performance than do individuals produced by mothers in better-quality environments (Mousseau and Fox 1998; Franzke and Reinhold 2012; Kelly et al. 2014). Our results are consistent with those of Magalhães et al. (2011) that show that the offspring of mothers reared on pepper, tomato or cucumber leaves had higher performances on these host species than do offspring of mothers reared on bean leaves, which is nevertheless considered as a host plant of high quality for mites (Agrawal 2000). Our study thus shows that maternal conditions strongly influence the performance of *T. urticae* individuals after a host plant shift could facilitate successful colonization of a novel host plant. Another hypothesis is that mothers from the tomato maternal environment have acquired a better resistance to the constitutive defense of tomato plants, which was transmitted to their offspring. These effects have been shown in *Daphnia* for which offspring that were challenged with the same bacterial strain to that of their mothers showed an increased immune response compared to offspring that were challenged with different strains from their mothers (Little et al. 2003). Similarly, in the milkweed bug arthropod, mothers can store cardenolide, a defensive compound from host plants, and transfer it into their eggs, which are then protected against predation (Newcombe et al. 2013).

The maternal conditions also affected the dispersal behavior of the offspring. Independently of the host plant on which their population evolved, *T. urticae* adults produced by mothers from the tomato environment left 1.3 times more the tomato testing environment than did adults from mothers of the cucumber environment. This pattern was independent of the sex of the individuals, and thus highlights that host plant acceptance, that is, a proxy of host plant suitability, by that is similar for males and females. Our results are consistent with the observation that the dispersal distance of offspring is strongly affected by maternal and grand-maternal environments (Bitume et al. 2014). We could thus infer that females reared on the tomato common environment produced adults with a dispersal capacity higher than those from females reared on the cucumber common environment, this capacity potentially being adaptive for searching for a more suitable host. An alternative hypothesis would be that mothers reared on tomato laid fewer eggs (personal observations) but were fitter and could disperse at a higher rate than did mothers reared on cucumber plants; this hypothesis cannot be checked as we did not measure individual fitness components in the maternal common environments. Independently of the underlying mechanism, dispersal of adults could prevent resource competition between parents and their offspring or prevent the induction of plant defenses, and thus be adaptive, as only high densities of *T. urticae* (more than 20 mites per leaf) can induce plant defenses (Karban and Carey 1984; Kant et al. 2004; Sarmento et al. 2011; Marinosci et al. unpublished manuscript).

### Environmental conditions can affect colonization of a novel host species

We detected that temporal blocks affected the juvenile mortality, the time spent as an adult in the tomato testing environment, and the female longevity and fecundity. These blocks may reflect one or several uncontrolled environmental variables, such as humidity, temperature, and light in the climate rooms. A previous experiment has indeed found that female fecundities and female longevities decrease with increased temperature (from 25 to 30°C; Riahi et al. 2013). The experimental, that is, environmental, conditions also affected the time at which adult mites left the testing environment, independently of their sex, and the longevity of adults that remained in the testing environment. Extrapolated to natural conditions, these results suggest that host colonization critically depends on environmental conditions by affecting directly female fitness and both male and female dispersal behavior.

## References

[b1] Agrawal AA (2000). Host-range evolution: adaptation and trade-offs in fitness of mites on alternative hosts. Ecology.

[b2] Bitume EV, Bonte D, Magalhães S, San Martin G, Van Dongen S, Bach F (2011). Heritability and artificial selection on ambulatory dispersal distance in *Tetranychus urticae*: effects of density and maternal effects. PLoS One.

[b3] Bitume EV, Bonte D, Ronce O, Olivieri I, Nieberding CM (2014). Dispersal distance is influenced by parental and grand-parental density. Proc. R. Soc. B.

[b4] Bolter CJ, Jongsma MA (1995). Colorado potato beetles (*Leptinotarsa decemlineata*) adapt to proteinase inhibitors induced in potato leaves by methyl jasmonate. J. Insect Physiol.

[b5] Dicke M, Dijkman H (1992). Induced defence in detached uninfested plant leaves: effects on behaviour of herbivores and their predators. Oecologia.

[b6] Ehrlich PR, Raven PH (1964). Butterflies and plants: a study in coevolution. Evolution.

[b7] Fellous S, Angot G, Orsucci M, Migeon A, Auger P, Olivieri I (2014). Combining experimental evolution and field population assays to study the evolution of host range breadth. J. Evol. Biol.

[b8] Fox CW, Wagner JD, Cline S, Thomas FA, Messina FJ (2011). Rapid evolution of lifespan in a novel environment: sex-specific responses and underlying genetic architecture. Evol. Biology.

[b9] Franzke A, Reinhold K (2012). Transgenerational effects of diet environment on life history and acoustic signals of a grasshopper. Behav. Ecol.

[b10] Fricke C, Arnqvist G (2007). Rapid adaptation to a novel host in a seed beetle (*Callosobruchus maculatus*): the role of sexual selection. Evolution.

[b11] Fritz RS, Simms EL (1992). Plant resistance to herbivores and pathogens. Ecology, evolution, and genetics.

[b12] Fry JD (1990). Trade-offs in fitness on different hosts: evidence from a selection experiment with a phytophagous mite. Am. Nat.

[b13] Gould F (1979). Rapid host range evolution in a population of the phytophagous mite *Tetranychus urticae* Koch. Evolution.

[b14] Kant MR, Ament K, Sabelis MW, Haring MA, Schuurink RC (2004). Differential timing of spider mite-induced direct and indirect defenses in tomato plants. Plant Physiol.

[b15] Kant MR, Sabelis MW, Haring MA, Schuurink RC (2008). Intraspecific variation in a generalist herbivore accounts for differential induction and impact of host plant defences. Proc. R. Soc. B.

[b16] Karban R, Carey JR (1984). Induced resistance of cotton seedlings to mites. Science.

[b17] Kavousi A, Chi H, Talebi K, Bandani A, Ashouri A, Naveh VH (2009). Demographic traits of *Tetranychus urticae* (Acari: Tetranychidae) on leaf discs and whole leaves. J. Econ. Entomol.

[b18] Kawecki T, Ebert D (2004). Conceptual issues in local adaptation. Ecol. Lett.

[b19] Kawecki T, Lenski RE, Ebert D, Hollis B, Olivieri I, Whitlock MC (2012a). Experimental evolution. Trends Ecol. Evol.

[b20] Kawecki T, Lenski RE, Ebert D, Hollis B, Olivieri I, Whitlock MC (2012b). The value of complementary approaches in evolutionary research: reply to Magalhães and Matos. Trends Ecol. Evol.

[b21] Kelly CD, Neyer AA, Gress BE (2014). Sex-specific life history responses to nymphal diet quality and immune status in a field cricket. J. Evol. Biol.

[b22] Kennedy GG, Helle W, Sabelis MW, Smitley DR (1985). Dispersal. Spider mites: their biology, natural enemies and control.

[b23] Kojima W, Fujii T, Suwa M, Miyazawa M, Ishikawa Y (2010). Physiological adaptation of the Asian corn borer *Ostrinia furnacalis* to chemical defenses of its host plant, maize. J. Insect Physiol.

[b24] Little TJ, O’Connor B, Colegrave N, Watt K, Read AF (2003). Maternal transfer of strain-specific immunity in an invertebrate. Curr. Biol.

[b25] Macke E, Magalhães S, Khanh HD-T, Luciano A, Frantz A, Facon B (2011). Sex allocation in haplodiploids is mediated by egg size: evidence in the spider mite *Tetranychus urticae* Koch. Proc. R. Soc. B.

[b26] Magalhães S, Matos M (2012). Strengths and weaknesses of experimental evolution. Trends Ecol. Evol.

[b27] Magalhães S, Fayard J, Janssen A, Carbonell D, Olivieri I (2007). Adaptation in a spider mite population after long-term evolution on a single host plant. J. Evol. Biol.

[b28] Magalhães S, Blanchet E, Egas M, Olivieri I (2009). Are adaptation costs necessary to build up a local adaptation pattern?. BMC Evol. Biol.

[b29] Magalhães S, Blanchet E, Egas M, Olivieri I (2011). Environmental effects on the detection of adaptation. J. Evol. Biol.

[b30] Mousseau TA, Fox CW (1998). The adaptive significance of maternal effects. Trends Ecol. Evol.

[b31] Newcombe D, Blount JD, Mitchell C, Moore AJ (2013). Chemical egg defence in the large milkweed bug, *Oncopeltus fasciatus*, derives from maternal but not paternal diet. Ent. Exp. Appl.

[b32] R Core Team (2013). R: A language and environment for statistical computing.

[b33] Riahi E, Shishehbor P, Nemati AR, Saeidi Z (2013). Temperature effects on development and life table parameters of *Tetranychus urticae* (Acari: Tetranychidae). J. Agr. Sci. Tech.

[b34] Sarmento RA, Lemos F, Bleeker PM, Schuurink RC, Pallini A, Oliveira MGA (2011). A herbivore that manipulates plant defence. Ecol. Lett.

[b35] Schoonhoven LM, Van Loon JJA, Dicke M (2005). Insect-plant biology.

[b36] Via S (1990). Ecological genetics and host adaptation in herbivorous insects: the experimental study of evolution in natural and agricultural systems. Annu. Rev. Entomol.

[b37] Wise MJ, Rausher MD (2013). Multiple-herbivore community: genetic correlations, diffuse coevolution, and constraints on the plant’s response to selection. Evolution.

